# Potential Role of Avian Populations in the Epidemiology of *Rickettsia* spp. and *Babesia* spp.

**DOI:** 10.3390/vetsci8120334

**Published:** 2021-12-17

**Authors:** Valentina Virginia Ebani, Francesca Mancianti

**Affiliations:** 1 Department of Veterinary Sciences, University of Pisa, Viale delle Piagge 2, 56124 Pisa, Italy; francesca.mancianti@unipi.it; 2 Centre for Climate Change Impact, University of Pisa, Via del Borghetto 80, 56124 Pisa, Italy; 3 Interdepartmental Research Center “Nutraceuticals and Food for Health”, University of Pisa, Via del Borghetto 80, 56124 Pisa, Italy

**Keywords:** migratory birds, avian population, ticks, *Rickettsia* spp., *Babesia* spp., zoonoses

## Abstract

Birds often are carriers of hard and/or soft ticks harboring pathogens of humans and veterinary concern. Migratory avian species, which cover long distance by their flight, may deeply influence the ticks’ distribution worldwide; in particular, they can introduce in a given geographic area new tick species and related tick-borne pathogens. Studies about the detection of tick-borne agents in birds are not numerous, whereas more attention has been turned to the presence of these microorganisms in ticks carried by birds. The present review focused on the role of avian populations in the epidemiology of rickettsioses and babesioses, which represent two severe problems for the health of humans and other mammals.

## 1. Introduction

Ticks are considered the most important vectors of microbial pathogens, zoonotic too, after mosquitoes [1]. These Arachnids can harbor more than one pathogen simultaneously and although Ixodidae are the most common vectors, Argasidae can also be involved, being able to transmit viruses, bacteria and, to a lesser extent, protozoan parasites such as *Babesia* [2]. Another relevant aspect of ticks’ parasitism is linked to the transportation of these Acharina, by affected hosts.

Birds with different lifestyles are, in fact, often carriers of ticks harboring pathogens, which can affect other animal species and humans. Tick infestation of birds is related to the rhythms of tick seasonal activity and birds’ migrations. Migratory birds, which cover long distances during their seasonal migrations, can introduce ticks and related tick-borne pathogens into new geographic areas [3].

Climatic changes, as well as consequent changing of environmental conditions, are factors that are influencing the tick distribution in several areas of the world. This feature is of capital importance for Ixodidae, which spend longer periods on hosts in respect to Argasidae. Birds are parasitized by both ticks’ Families [4] and play an important role in the dispersal of tick-borne diseases, acting both as cyclic hosts and as carriers of viruses, bacteria, and protozoa.

The role of avian populations in the epidemiology of some pathogens transmitted by hematophagous arthropods has been defined. For example, several investigations have clarified the involvement of wild birds in the cycle of *Borrelia* spp. and West Nile virus [5]. On the other hand, some arthropod-borne agents have been found in ticks removed from birds, but several aspects related to these microorganisms are currently not clear: duration of pathogens’ presence in blood, target organs, potential pathogenicity for birds.

The present review focused on the role of birds in the epidemiology of *Rickettsia* spp. and *Babesia* spp., which are some of the most important bacterial and protozoan agents of veterinary and human concern, because they may cause diseases characterized by mild or severe clinical forms in different mammal species, including humans.

## 2. *Rickettsia* spp.

### 2.1. Etiology

The genus *Rickettsia* comprises obligate intracellular Gram-negative bacteria, whose sizes range from 0.3–2.0 µm. These bacteria multiply by binary fission in the cytoplasm of eukaryotic host cells and consequently, they must be cultivated in tissue culture; no axenic media has been formulated for their cultivation. *Rickettsiae* are difficult to stain with ordinary bacterial stains but, conveniently, are stained by the Gimenez method.

Members of this genus are currently classified into four groups: the typhus group (TG) with *R. typhi* and *R. prowazekii*; the spotted fever group (SFG) with 21 species; *R. bellii* group; *R. canadensis* group. Many other isolates exist, but they are not fully characterized [6].

*Rickettsiae* have a life cycle typically involving hematophagous arthropod vectors and vertebrate hosts. They enter the host cells by phagocytosis and escape from the phagocytic vacuole into the host cytoplasm to evade phagosome-lysosome fusion, replicate within the host cytoplasm, and exit from the host cell by actin-mediated motility (SFG) or lysis of host cells (TG).

TG *rickettsiae* are responsible for severe human diseases: murine typhus and epidemic typhus are caused by *R. typhi* and *R. prowazekii*, respectively.

SFG members are the cause of different diseases, often serious, that may affect both humans and other mammals. Among them, *R. conorii* is the most widespread SFG species in the countries of Mediterranean basin, responsible for the Mediterranean spotted fever (MSF) that affects humans, but also dogs [7]. Other SFG species are present worldwide, often associated to human clinical cases, with different distribution in relation to geographic areas. Among them, *R. helvetica* has been reported in several European countries, other than in Africa and Asia. It is associated in humans to aneruptive fever, headache, arthralgia, myalgia, perimyocarditis, whereas its pathogenic role in animals is not clarified [8]. Similarly, *R. monacensis* causes diseases in humans, but its potential pathogenicity for animals is not known. It has been reported mainly in Europe, even though reports come from South Korea, Algeria, and USA [9]. *Rickettsia massiliae* can affect dogs and mainly humans, in which causes a febrile illness with rash, inoculation eschar and neck lymphadenopathy. It is distributed in tropical African countries, southern Europe, and USA [10,11]. *Rickettsia monacensis* has been reported in Europe, Asia, northern Africa, and USA, as well as *R. aeschlimannii* in southern Europe, several African countries and central Asia; both species induce clinical pictures similar to MSF in affected persons [10]. *Rickettsia slovaca* and *R. raoultii*, present in Europe and Asia, are associated to the human disease tick-borne lymphadenopathy (TIBOLA), *Dermacentor*-borne necrosis erythema and lymphadenopathy (DEBONEL) and scalp eschars neck lymphadenopathy (SENLAT) [10]. African-tick-bite fever (ATBF) is caused by *R. africae* and is frequently diagnosed in Europe in patients returning from African endemic areas [10].

### 2.2. Rickettsia spp. in Birds and Their Ticks

Studies carried out to verify the role of avian populations in the epidemiology of rickettsiosis mainly regard investigations about the presence of rickettsiae in ticks collected from birds. Otherwise, surveys about *Rickettsia* infection directly in birds are scanty (Table 1).

During an investigation carried out in Cyprus from 2004 to 2006 by Ioannou and coworkers [12], 131 blood pools were obtained from 557 wild birds belonging to 51 avian species. Among them, four (3%) (common moorhens *Gallinula chloropus* and American flamingos *Phoenicopterus ruber*) were PCR-positive for *Rickettsia* spp.; furthermore, ticks and lice were removed from the tested animals and *Rickettsia* spp. DNA was found in three *Ixodes vellantoi* [12].

During the 2009 and 2010 spring migration seasons, 2064 northward-migrating passerine songbirds were examined for ticks at Johnson Bayou, Louisiana (USA) [13]. A total of 91 ticks were collected from 35 songbirds and they were identified as *Haemaphysalis juxtakochi* (42%), *Amblyomma longirostre* (24%), *Amblyomma nodosum* (19%), *Amblyomma calcaratum* (12%), *Amblyomma maculatum* (2%), and *Haemaphysalis leporispalustris* (1%). Most of the identified ticks were exotic species originating outside of the United States. Two genotypes, found in 28 ticks, were included in the *Candidatus Rickettsia* amblyommii clade, 2 genotypes, detected in 14 ticks, clustered with *Rickettsia* sp. “Argentina”, and 2 genotypes, found in 27 ticks, exhibited sufficient genetic divergence to possibly constitute a new rickettsial genotype. Moreover, blood samples were collected from the 35 birds and 20 rickettsial strains were detected. Analyses of the detected rickettsial DNA identified 18 strains identical to *Rickettsia* sp. “Argentina” and to *Rickettsia* endosymbiont of *Amblyomma maculatum*, one strain different only for 2 base pairs, while the quality of the last strain did not allow the identification.

Interesting results were observed comparing the presence of rickettsiae in ticks and birds. In fact, 14 ticks were not infected and were found on infected birds, 18 were infected, but found on noninfected birds, and 6 were not infected and were found on non-infected birds. Furthermore, only in 5 cases, the rickettsia found in the tick from a given bird was the same as that detected in that bird, while in the remaining cases the rickettsia detected in the ticks did not correspond to the strain from the bird [13].

Successively, Hornok et al. [14] collected, in spring and autumn migration periods of 2013, blood specimens from 128 wild birds in Hungary. Five robins (*Erithacus rubecula*) and one dunnock (*Prunella modularis*), for a total prevalence of 4.7%, were PCR-positive with *R. helvetica*. All ticks on the body of each examined bird were removed and analyzed. A total of 140 ticks were collected from 68 birds; *Ixodes ricinus* was found on most of the tick-infested birds (92.6%) and it was the most abundant tick species (86.4%). *Haemaphysalis concinna* was present on 11.8% of the infested birds; four animals were co-infested with *I. ricinus* and *H. concinna*; one wood warbler (*Phylloscopus sibilatrix*) carried a *Hyalomma* larva. Of the 140 ticks, 61, *I. ricinus* and *H. concinna*, were PCR-positive for *R. helvetica* and 11, all *I. ricinus*, for *R. monacensis*. The no-correlation between the PCR results testing birds and their ticks is interesting. In fact, among the six birds resulted positive for *R. helvetica*, no ticks were found on four of them and one PCR-negative tick was found on another. The remaining *R. helvetica* positive birds had 11 ticks, of which two resulted PCR-positive. On the other hand, 59 ticks positive for *R. helvetica* were removed from 38 PCR-negative birds.

The authors supposed that rickettsiaemia in avian hosts persists after the detachment of the ticks, as corroborated by the no-findings of ticks on the majority of *R. helvetica* positive birds. Moreover, they hypothesized that the transmission of rickettsiae is possible from ticks to birds, whereas the transmission from birds to ticks rarely occurs, as suggested by the fact that among 11 ticks removed from a rickettsiaemic bird, only two were PCR-positive for *R. helvetica*. However, the finding of PCR-positive ticks removed from PCR-negative birds could be related to potentially intermittent rickettsiaemia of the animals [14]. Moreover, PCR-negative results could not correspond to absence of rickettsiae in the tested samples, because PCR is not always able to detect DNA of pathogens; in fact its sensitivity is in relation to the assay’s protocol, type of samples, and amount of pathogens present in the specimens [19].

Feral pigeons (*Columba livia domestica*) were found infected by *Rickettsia* spp. with a prevalence of 5.95% in central Italy. In fact, DNA of these pathogens was found in spleen samples collected from five pigeons captured in urban/peri-urban areas [15], suggesting that feral pigeons, which are traditionally considered a hazard for the human health as source of pathogens excreted with feces, such as salmonellae and chlamydiae, may be also involved in the epidemiology of rickettsiosis.

Few studies documented the exposure of birds to rickettsiae by the detection of specific antibodies. A serological survey was carried out in pheasants (*P. colchicus*) to verify the occurrence of antibodies against some tick-borne pathogens. All the tested pheasants lived in an area of central Italy with conditions favorable for ticks’ diffusion, such as a rich vegetation and various large and small mammals [17]. The 17.75% of the examined animals were positive to *R. conorii* antigen [17]. The detected prevalence could include antibodies against *R. conorii*, as well as other SFG rickettsiae due to the common antigens [7]. In fact, several SFG rickettsiae, mainly *R. conorii, R. helvetica, R. massiliae, R. slovaca, R. aeschlimannii, R. africae, R. monacensis, R. roultii* were circulating in Italy at the sampling time, as observed in molecular investigations in animals and ticks [17].

A previous serological survey detected antibodies to *R. africae* antigen in 35% in ostriches (*Struthio camelus*) living in farms in Zimbabwe [18]. Additionally in this case, the detected antibodies could be due to other SFG rickettsiae, such as *R. conorii* that circulated in the area in that period. However, the authors supposed that the examined ostriches were exposed to *R. africae*, because this rickettsia had been identified in *Amblyomma hebraeum* that usually parasitized ostriches in Zimbabwe, whereas *R. conorii* was traditionally associated to *Rhipicephalus* sp. that were not known to feed on these birds.

Most numerous data available in literature are focused on the presence of DNA rickettsiae in hard and soft ticks removed from birds.

Santos-Silva and coworkers [20] analyzed ticks removed from wild birds in Portugal from 2002 to 2004, but they did not verify if the animals were rickettsaemic. However, three *Hyalomma marginatum* had rickettsial DNA exhibiting nucleotide sequence of *gltA* (citrate synthase) and *ompA* genes 100% similar to *R. aeschlimannii*. One *Rhipicephalus turanicus* had a rickettsial agent showing 100% homology to *R. massiliae*, as well as one *I. vellantoi* had a rickettsia 100% homologous to *R. helvetica*.

A survey investigated the tick infestation in populations of the migratory passerine bird *Riparia riparia*, the sand martin, in northwest England in June 2009. A total of 194 birds were sampled and ticks removed from infested birds and all ticks were identified as females of *Ixodes lividus.* A single *Rickettsia* spp., designated as *Rickettsia* sp. IXLI1, was detected in 100% of the ticks examined by PCR. Partial sequences of 17-kDa and *ompA* genes showed greatest similarity to the strain *Rickettsia* sp. TCM1, etiological agent of the Japanese spotted fever-like illness, previously described in Thailand. Moreover, phylogenetic analysis showed that the strain *Rickettsia* sp. IXLI1 perfectly fitted into a group containing *Rickettsia japonica*, *Rickettsia* sp. strain Davousti, and *Rickettsia heilongjiangensis* [21].

A molecular survey, carried out in autumn 2010, involved 2,793 migratory birds captured by nets in a ringing station located in northern Italy; in particular, the birds were captured in a woodland zone (Arosio, Como, Italy) located ~30 km north of Milan, one of the most populated and interconnected areas in Europe [22]. The animals were checked for the presence of ticks and 251 nymphs and larvae of *I. ricinus* were collected and examined by PCR. Twenty-five (10%) ticks were positive for *Rickettsia* spp.: 1 was removed from a chaffinch (*Fringilla coelebs*), 1 from a common blackbird (*Turdus merula*), 2 from robins (*E. rubecula*), 5 from redwings (*Turdus iliacus*), 16 from song thrushes (*Turdus philomelos).* The detected amplicons were reportable to *R. monacensis* and *R. helvetica*, but it is mainly interesting the detection of *Candidatus Rickettsia mendelii* [22] that was recently described as a non-SFG *Rickettsia* in *I. ricinus* from Czech Republic [23]. This finding is the typical example of the potential role of avian population in carrying infected ticks from one geographic area to another.

An interesting survey was carried out by Wallmenius et al. [24] on many samples. Ticks were collected from a total of 14,789 birds during their seasonal migration northwards in spring 2009 and 2010 at bird observatories on two Mediterranean islands: Capri (Italy) and Antikythera (Greece). All ticks were subjected to RNA extraction followed by cDNA synthesis and individually assayed with a real-time PCR targeting the *gltA* gene. For species identification of *Rickettsia*, multiple genes were sequenced. Three hundred and ninety-eight (2.7%) of all captured birds were tick-infested; some birds carried more than one tick. A total number of 734 ticks were analyzed and 48% were *Rickettsia*-positive; 96% were infected with *R. aeschlimannii*, and 4% with *R. africae* or unidentified *Rickettsia* species. The predominant tick taxon, H. marginatum sensu lato constituted 90% of the ticks collected. The remaining ticks were *Ixodes frontalis*, *Amblyomma* sp., *Haemaphysalis* sp., *Rhipicephalus* sp., and unidentified ixodids. Most ticks were nymphs (66%), followed by larvae (27%), and adult female ticks (0.5%). The majority (65%) of ticks were engorged and nearly all ticks contained visible blood.

A similar screening for multiple tick-borne pathogens in *I. ricinus* ticks collected from wild birds in Denmark, during spring and autumn migration seasons in 2016, found that 10.6% of the examined ticks were PCR-positive for SFG *rickettsiae* [25].

*Rickettsia* sp. DNA were found in 12.3% of *I. ricinus* removed from wild birds in Switzerland during breeding periods and fall migrations from 2007 to 2010. In particular, *R. helvetica* and *R. monacensis* DNA were detected with prevalences of 10.5% and 0.4%, respectively [26]. More in detail, larvae (13.6%) were more infected than nymphs (10.1%); the prevalence found in ticks feeding on migrants (11.9%) was lower than in ticks feeding on breeding birds (17.3%). The common blackbird *T. merula* (25%) and the tree pipit *Anthus trivialis* (23.3%) were the species most frequently infested with *Rickettsia*-infected ticks.

The reservoir competence of tree pipits for *R. helvetica* was suggested by the authors, in this study, since 5.8% of tree pipits were infested by at least two infected larvae and larvae (17.3%) were more infected than nymphs (11.8%). However, *rickettsiae* could be transmitted transovarially to larvae, thus the reservoir competence of avian hosts should be otherwise demonstrated. Furthermore, during the same study it was observed that larvae feeding with nymphs (16.3%) showed a very similar infection prevalence as larvae feeding alone on host (without nymphs) (17.9%), suggesting that co-feeding transmission is not the main means of transmission and that birds may be the source of infection. The same authors concluded that *T. merula* are also important in the life cycle of *rickettsiae*, since they are the species carrying the most frequently infected [26].

Similarly, the reservoir competence of some avian genus for *Rickettsia* spp. was suggested by some authors. For example, Elfving et al. [27] found one European Blue Tit (*Parus caeruleus*) which carried seven ticks of which six were positive for *R. heilongjiangensis*-like *Rickettsia*. The authors supposed that the bird was rickettsiaemic and that the ticks had acquired the bacteria from its blood.

On the other hand, there were also several examples of multiple ticks on a single bird where only one of the ticks was infected with *Rickettsia*, for instance a European robin that carried 27 ticks, only one of which was positive for *R. helvetica* [27].

A potential role of some avian species as reservoirs was supposed also by Spitalska et al. [28], who detected rickettsiae in 44.0% larvae and 24.5% nymphs of *I. arboricola* collected from great tits (*Parus major*), marsh tits (*Poecile palustris)*, and Eurasian nuthatches (*Sitta europaea*). Rickettsiae-positive *I. ricinus* larvae (13.7%) were collected from *P. major*, *Cyanistes caeruleus* (Euraian blue tits) and *S. europaea*, and 2.6% of nymphs from robins (*Erithacus rubecula*) and dunnocks (*Prunella modularis*) [28].

Recently, Rollins and coworkers [29] collected ticks from 151 birds captured on Ponza Island (south Italy), which represents an important migratory stopover off the western coast of Italy. In total, 16 captured birds carried ticks from four tick species: *Hyalomma rufipes* (n = 14), *Amblyomma variegatum* (n = 1), *Amblyomma* sp. (n = 1), and *Ixodes ventalloi* (n = 2). All specimens were either larvae (n = 2) or nymphs (n = 16). *R. aeschlimannii* was detected in six of the 14 collected *H. rufipes* ticks and the singular *A. variegatum* nymph tested positive for *R. africae*.

Ticks carried by wild birds may be source of rickettsiae at high altitudes, too, as demonstrated by a study conducted in the Tropical Andes in Colombia from 2015 to 2019. Ticks were collected from birds at elevations ranging from 178m to 3845m. *Rickettsia* DNA was found in 11% of the examined ticks, which were removed from six resident bird species and one migratory boreal species. *Rickettsia amblyommatis* was found in *A. longirostre, Amblyomma varium* and *Ixodes* sp. (closely related to *Ixodes auritulus*) collected from resident avian species. *Candidatus Rickettsia colombianensi* was detected in *Amblyomma dissimile* taken from the migratory bird *Opornis agilis*, whereas a possible novel agent closely related to *Candidatus Rickettsia tarasevichiae* and *R. canadensis* was detected in *Ixodes* sp. (closely related to *Ixodes tanuki*) [30].

*R. amblyommatis*, as well as *R. bellii*, was also found in *A. longirostre* and *A. calcaratum* recovered from wild birds in Brazil [31,32]. *Amblyomma* spp. ticks infesting wild avian population in Brazil resulted carriers of *Rickettsia rhipicephali, Rickettsia parkeri*, and *Candidatus* Rickettsia amblyommii, too [33].

Cold geographic areas are not free from this problem. A *Rickettsia*-like microorganism was isolated from ticks of the species *Ixodes uriae* collected from penguins (*Pygoscelis papua*) bred on Mayes Island, Kerguelen Archipelago, French Subantarctic Territories [34]. More recently, a king penguin (*Aptenodytes patagonicus*) recovered and dead in a rehabilitation center in South Africa resulted infected by a *Rickettsia*-like organism. Post-mortem blood smears of this animal showed pleomorphic rickettsia-like inclusions within the cytoplasm of erythrocytes. It was supposed that the animal died because of bacterial enteritis with complicating septicemia to which the rickettsial agent may have been related [16].

Even though hard tick species result the main hematophagous vectors of *rickettsiae* among birds, soft ticks (*Argasidae*) seem to be involved in the transmission of these pathogens, too.

*Rickettsia hoogstraali* was found in 41% of *Argas* (*Persicargas*) *giganteus* ticks removed from wild birds recovered in a wildlife rehabilitation center in Arizona. However, the blood samples of birds, from which positive ticks were collected, resulted PCR-negative for *Rickettsia* spp. [35].

*Rickettsia fournieri* sp.nov., a novel SFG species, was isolated from an *Argas lagenoplastis* tick collected from the nest of a *Petrochelidon ariel* (fairy martin) in Australia in 2013 [36].

*Ornithodoros sawaii* and *Ornithodoros capensis* were assessed for the presence and identification of *rickettsiae* by Kim et al. [37] in Gugul Island, Republic of Korea. Ticks collected from samples of nest litter and soil from seabird nests were identified individually by morphological techniques, and species confirmed by sequencing of the mt-rrs gene. A total of 134 soft ticks belonging to *O. sawaii* and *O. capensis* were collected; *Rickettsia lusitaniae* DNA was detected and identified in 11 (8.8%) O. sawaii ticks collected from nest litter and soil of the Japanese murrelet (*Synthliboramphus wumizusume*) [37].

*Rickettsia* sp. was first detected in seabird soft-bodied ticks, *Carios capensis* and *Carios sawaii* in Japan by Kawabata and coworkers from 2003 to 2005 [38]. Ticks were collected from colonies of streaked shearwaters (*Calonectris leucomelas*) and black-footed albatross (*Diomedea nigripes*). The sequence of this *Rickettsia* strain was identical to that of a *Rickettsia* strain (scc31) previously found in *C. capensis* in South Carolina, U.S.A. This finding suggested that an environmental circulation had consisted among microorganisms, ticks, and long-distance migratory seabirds around the Pacific Ocean [38].

*Carios capensis*, well known as a tick of seabirds, often infests the nests of brown pelicans (*Pelecanus occidentalis*) and other ground nesting birds along the coast of South Carolina. Reeves et al. 2006 [39] collected ticks from a pelican rookery on Deveaux Bank, South Carolina and *R. felis* and two undescribed *Rickettsia* spp. DNA were detected. *Rickettsia felis*, which causes a murine typhus like human disease, is related to cat populations and fleas *Ctenocephalides felis*. The susceptibility of pelicans to this rickettsia as well as its source in *C. capensis* were not defined. It was supposed that pet dogs, which in South Carolina are frequently infested with *C. felis*, could be the source of fleas infected by *R. felis*. In fact, even though pets are not allowed on Deveaux Bank, dog footprints were noted by the researchers near human footprints among the pelican nests [39].

From 2012 to 2014 soft ticks of the species *C. capensis* were collected from yellow-legged gull (*Larus michaellis*) nests in Algeria [40]. Sequences of the *gltA* gene of the detected rickettsial DNA suggest that the microorganism is a novel SFG *Rickettsia* strain, probably a strain of *R. hoogstraalii*. Based on other findings, this species seems to be the most common rickettsial species infecting *C. capensis* ticks, confirming that this hematophagous arthropod likely represents a major host reservoir for the bacteria. An unclassified rickettsial lineage genetically related to *R. hoogstraalii* was also detected in *C. capensis* in Réunion (Europe) and the Tromelin Islands, showing that two distinct *Rickettsia* spp. strains cocirculate in these tick populations [41].

During the same survey by Dietricht et al. [41] on Réunion Island, *R. bellii* was detected in 2 *C. capensis* ticks collected within the same nest of the wedge-tailed shearwater (*Puffinus pacificus*).

*R. bellii* has been most frequently documented in the Americas, but recent studies have detected the presence of closely related *R. bellii* strains also in Australia [42] and Europe [43]. The presence of *Rickettsia* agents on Réunion Island, considered as a remote island, is a further suggestion of the role of bird hosts in the dispersal of ticks and their associated infectious agents over large distances.

## 3. *Babesia* spp.

### 3.1. Etiology

*Babesia* spp. are apicomplexan parasites that, together with *Theileria*, belong to piroplasms. They are so called for their pear-shaped morphology, although oval or round forms can be detected, and appear as small endocellular organisms (1–5 μm in length) [44]. These parasites can be differentiated by pigment forming *Plasmodium* and *Haemoproteus*, lacking pigment into erythrocytes. *Theileria* differs from *Babesia* for its ability to form schizonts, to colonize lymphocytes, besides red cells and to transmit only transstadially in ticks. Furthermore, babesiae are classified in *Babesia* sensu stricto (transstadial and transovarial transmission) and *Babesia* sensu lato (transstadial transmission only). Life cycle of *Babesia* consists in asexual multiplication in red blood cells of the vertebrate host and in gametogony followed by sporogony in salivary glands of ticks that have acquired the infection during the blood meal [45,46]. More than 100 parasite species have been described in animals. They are often pathogenic, mostly for domestic animals and human beings, showing a strong economic impact, negatively influencing animal welfare and public health. Babesiosis in wildlife can be fatal, too when associated with stressful management practices [45]. Mammals and birds are involved in life cycles as vertebrate (intermediate) hosts although the specificity of babesia for vector ticks (final hosts) has not been fully elucidated [47].

*Babesia* spp. accomplish merogony into red blood cells of birds and gametogony and sporogony in both Ixodidae and Argasidae ticks, [48,49]. Argasidae, mostly *Ornithodoros* seem to be involved in *Babesia* transmission in colonial birds nesting on ground or near rocks, whose crevices would represent breeding sites for these invertebrate hosts. The occurrence of *Ornithodoros capensis* on African penguins seems to be strongly related with *Babesia* infection higly supporting the hypothesis that this tick would be the main vector of the parasites [50,51]. These findings have been corroborated by the occurrence of *Babesia* DNA in this argasid species from New Zealand too [52].

### 3.2. Babesia in Birds and Their Ticks

The first record of Babesia in birds goes back to the beginning of last century and the parasite was identified as *Sogdianella moshkovskii* [53], then modified in *B. moshkovskii* [54]. Sixteen species of avian *Babesia*, namely *B. moshkovskii*, *B. kazachstanica*, *B. uriae*, *B. kiwiensis*, *B. ardeae*, *B. frugilegica*, *B. emberizica*, *B. shortti*, *B. balearicae*, *B. rustica*, *B. bennetti*, *B. mujunjumica*, *B. peircei*, *B. poelea*, *B. krylovi*, and *B. ugwidiensis* have been recognized to date [48,55,56,57], along with other *Babesia* spp. although, *B. poelea* is suggested to be a synonym of *B. peircei*, able to infect seabirds from several orders [48,58]. Each species would be host specific at the family level [59]. Since only a limited number of morphological characters are available (chromatin of merozoites of *B. peircei*, *B. krylovi, B. poelea*, *B. uriae*, and *B. bennetti* has a distal position, when compared with other species whose nuclei are in a proximal position), species cannot always be differentiated morphologically [60]. 

Some of these species have been molecularly characterized [58], indicating that these avian parasites are not monophyletic, and belong to three paraphyletic groups namely *Peircei*, *Kiwiensis*, and *Bennetti*. *Peircei* group is distributed worldwide in aquatic birds and comprehends *B. ardeae, B. poelea, B. peircei, B. ugwidiensis, B. uriae* and other not identified *Babesia* spp. *Kiwiensis* group is reported from the Pacific Ocean, only and *Bennetti*, containing *B. bennetti* as a unique species, has been registered only in Mediterranean Sea.

Small subunit ribosomal RNA gene sequences are available only for *B. poelea, B. uriae, B. bennetti, B. kiwiensi, B. ugwidiensis*, and *B. uriae*, but not for *B. peircei*, so the piroplasms found in healthy little penguins from Australia affected only by round forms molecularly closely related to *B. poelea* and *B. uriae*, could be *B. peircei*, confirming the specificity of this species for penguins [61], although, as above reported, *B. poelea* was suggested be a synonym of *B. peircei*, considered as a generalist parasite species, infecting seabirds, belonging to different orders. Furthermore, New Zealand seabirds were found infected by novel variants of *B. poelea*-like protozoa (named genotypes I and II) and by a *B. kiwiensis*-like piroplasm. All the three variants were isolated from gannets and gulls, whilst terns scored positive for *B. poelea*-like genotype I *B. kiwiensis*-like parasites, only [52], suggesting a lack of stratification by avian species.

The prevalence of infection in birds is low [62,63] and the finding of *Babesia* in different unrelated avian species would suggest that such infections have followed many independent colonization events [56,62].

The pathogenicity of *Babesia* spp. for birds is still unclear [59], and *B. shortii* and *B. uriae* seem the only pathogenic avian piroplasms. *B. uriae* has been identified in dead murres, showing associated lesions [56], while *B. shortii* has been described in Falconidae that are probably the only susceptible species and is considered a pathogenic piroplasm in its avian hosts [64]. For these reasons *B. shortii* is considered as the most important avian *Babesia* species, both from a conservation and veterinary standpoint [48]. However, *B. peircei* was isolated from diseased penguins, affected by other haemoparasites, too: these piroplasms seem to have contributed to the death of a king penguin [65]. Infected penguins are reported to show sometimes mild anaemia, leucocytosis, and impairment of hepatic function [66,67]. Thus, it is assumed that these protozoan species would contribute to the overall morbidity and mortality when other pathogens occur. Furthermore, *B. ugwidiensis* was reported from young cormorans showing high mortality rates and with parasite stages in blood. However, a relationship between the occurrence of high parasite load and mortality is still debated [65].

The hypothesis of pathogenic actions by these piroplasmic species is supported by the good response to antibabesial therapy in falcons treated with imidocarb dipropionate [64] and in African penguins and cormorants empirically administered with primaquine [68]. Avian *Babesia* spp. are listed in Table 2.

### 3.3. Carriage of Zoonotic Babesia Infected Ticks

Ticks recovered from avian species result infected by different *Babesia* spp., however, in the present study attention will be paid to zoonotic agents, only.

The role of migratory birds in spreading ticks across the Europe has recently been reviewed by Buczek et al. [3]. Birds not migrating and/or migrating are carriers of several ticks along with several tick pathogens and would play a role in spreading such agents [3,77]. Transport of tick-borne pathogens from one endemic area to another could have an impact, although the pathogens already occur there, by spreading new strains to new areas, too [78]. Birds usually bear larval and nymphal stages of *Ixodes ricinus* at a latitude 58°N, and *H. marginatum* in areas below 42°N [3], furthermore *Haemaphysalis concinna* is the second more prevalent ixodid species isolated from birds in Central Europe [79] and has been recently recognized to contain *Babesia* sp. (related to *B. crassa*) and *B. microti* DNA [80].

Tickborne zoonotic protozoa belonging to *Babesia* genus, namely *B. divergens, B. microti* and *B. venatorum* (formerly *Babesia* sp., EU1) are the most involved species [81]. However, *B. duncani* (*Babesia* sp. WA1), *B. crassa*-like, *Babesia* sp. KO, *Babesia* sp. CN1 (*Babesia* sp. XXB/HangZhou) [82] and *B. odocoilei* [83] have been recently included. *B. divergens* mostly occurs in Europe [84,85].

Zoonotic piroplasms are mostly transmitted by *Ixodes* ticks [86,87]. The vectors in Europe are *I. ricinus*, while in Asia and in America *Ixodes persulcatus* and *Ixodes scapularis*, respectively would occur [88]. Interestingly, *B. microti* transmitted by *I. ricinus* is not common in Europe, being reported in few cases per year, while, when transmitted by *I. scapularis* this piroplasm is responsible for several hundred cases per year [89].

Human babesiosis is considered as an emergent worldwide disease, potentially life threatening and severe, mostly for immunocompromised people. Clinical signs of *B. microti* infection range from asymptomatic to fulminating, fatal disease. The most common symptoms are fever, fatigue, malaise, anorexia, and myalgia. Immunocompromised patients would show severe babesiosis characterized by anemia, acute respiratory distress syndrome, disseminated intravascular coagulation, congestive heart failure, renal failure, and coma. *B. divergens* is reported mostly in asplenic patients from Europe and cases appeared severe, with a fulminant course and parasitaemia 80%. Babesiosis by *B. venatorum* from Europe is more severe than from China, where the course of disease appears milder. The clinical cases caused by *B. duncani* are less than 20 and symptomatology is similar to those of *B. microti* [44]. Finally, babesiosis by *B. odocoilei*, until today, has been described in two spleen-intact patients from USA, only, showing night sweats, chills, fevers, profound fatigue, increased thirst, muscle aches, and sleep disturbance [90].

Questing larvae are usually found on the ground, so ground-foraging birds (Passeriformes and Galliformes) are more frequently involved in hard ticks’ life cycle. Furthermore, migrating birds can shed engorged ticks to stopover sites, able to colonize further hosts which will carry them over longer distances [3].

Birds migrating southwards have high rates of infestation and *I. ricinus* is the predominant tick species [91]. For these reasons, epidemiological studies from Europe deal with identification of *Babesia* spp from *I. ricinus.* Passeriformes seem more frequently involved in carriage of infected ticks. *B. divergens* and *B. microti* DNA were found in specimens of *I. ricinus* mostly on blackbirds from Germany [92,93]. The same piroplasm species along with *B. venatorum*, was identified in ticks on Passeriformes from Latvia [94]. *B. venatorum* DNA has been identified in nymphs carried by *Turdus philomelos* in Northwestern Russia [95] and in Norway by passerine birds [78], while *B. microti* and *B. venatorum* infected both larval and nymphal stages of *I. ricinus* from Sweden [96]. Furthermore, DNA of *B. microti* was detected in *Ixodes granulatus* found on *Emberiza spadocephala* in Taiwan [97].

Similar studies from North America dealing with *I. scapularis*, revealed *B. microti* [98] while *B. odocoilei* was identified in ticks from Canada, occurring on songbirds [99,100,101]. The occurrence of a pathogen in a tick found on a host does not demonstrate in fact that the tick is the vector of the pathogen, and data would be misleading when the presence of pathogens has not been ascertained in the host [47].

However, for *Babesia* organisms the knowledge of vector identity is still incomplete and there are a lot of studies concerning the detection of parasite DNA in vectors, compared with properly controlled transmission studies to evaluate the vector competence of ticks [89].

### 3.4. Carriage of Zoonotic Babesia by Infected Birds

The role of birds as reservoir of zoonotic piroplasms has not been fully elucidated. The reservoirs can be identified by comparing the infection rates of questing preadult stages with larvae feeding on the hosts. When larvae show a higher infection prevalence it is reasonable to assume that the host is a reservoir [25], mostly when the pathogen fails to be transmitted transovarially, as in case of *B. microti* [92,98]. *B. divergens* and *B. venatorum* are, conversely, transmitted both transstadially and transovarially [102]. Furthermore, since the life cycle is quite complex, the hypothesis of a route of transmission by cofeeding should be ruled out [92].

Birds are considered as reservoir for zoonotic babesiae [92], namely *B. microti, B. divergens* and *B. venatorum.* These features have been confirmed by the ability to infect more than ten larvae of *I. scapularis* with *B. microti* showed by different avian species [98]. 

## 4. Conclusions

Birds have the capacity of flying across large intercontinental areas in short periods of time to exploit seasonal opportunities for food supplies and breeding habitats [103]. They fly along migration routes that connect several geographic areas. Numerous flyways are known in all continents. Millions of wild birds are flying from some areas to others twice a year in just a few weeks, and during this movement they can carry pathogens which may affect other birds, as well as humans and other mammals. Moreover, during their intercontinental movements birds can also carry hematophagous arthropods harboring microorganisms. The pathogens’ transmission continues in the stopover areas where numerous birds of different species are concentrated. The migratory routes, thus, can be considered a relevant cause of spreading of a given pathogen from an initial site of contagion to new remote areas [104].

Rickettsiae are tick-borne microorganisms with different distribution in relation to the geographic areas and are frequently the cause of severe human diseases. Migratory birds, which are often involved in the epidemiology of tick-borne pathogens, have been proven to be carriers of ticks harboring several *Rickettsia* species worldwide. In some cases, in relation to the species of ticks, rickettsiae and birds, high altitudes and low temperatures seem to not reduce the risk of circulation of arthropods and bacteria in avian populations [30,34].

Through flight of birds, migratory routes connect geographic areas with different climatic features and allow ticks and their related pathogens to arrive in new environments. Resident birds, that live in the same place throughout the year, may act as carriers of infected ticks, too. Among them, synanthropic species, such as pigeons, frequently live in urban and peri-urban areas representing a serious risk for humans not only as source of salmonellae or chlamydiae, but also tick-borne pathogens [15].

However, most of the data available in literature prove that avian species are carriers of *Rickettsia*-infected ticks, but only few studies have been carried out to verify the rickettsial infection in these animals. Further investigations are necessary to clarify this concern because the finding of *Rickettsia* DNA in ticks could be simply related to the presence of the pathogens in the gut blood reaming from the last feeding on host [13].

Tick-borne avian protozoa such as *Babesia* spp. do not represent a threat for public health, however birds can carry early stages of hard ticks infected with zoonotic species—although only a few studies dealing with the vector competence of ticks have been accomplished [89]. However, birds are considered as reservoir for zoonotic piroplasms, and this feature has been demonstrated for *B. microti* in different birds’ species [98]. For these reasons further studies are needed to fully evaluate the impact of avian species in the epidemiology of such pathogens.

## Figures and Tables

**Table 1:** *Rickettsia* agents detected in different avian species.

Bird Species	Common Name	Test (Sample)	*Rickettsia* Agent Detected	Geographical Distribution	Reference
*Gallinula chloropus*	Common moorhen	PCR (blood)	*Rickettsia* sp.	Cyprus	[12]
*Phoenicopterus ruber*	American flamingo	PCR (blood)	*Rickettsia* sp	Cyprus	[12]
*Hylocichla mustelina*	Wood thrush	PCR (blood)	*Rickettsia* sp. “Argentina”/REA	Louisiana (USA)	[13]
*Passerina ciris*	Painted bunting	PCR (blood)	*Rickettsia* sp. “Argentina”/REA	Louisiana (USA)	[13]
*Wilsonia citrina*	Hooded warbler	PCR (blood)	*Rickettsia* sp. “Argentina”/REA	Louisiana (USA)	[13]
*Passerina cyanea*	Indigo bunting	PCR (blood)	*Rickettsia* sp. “Argentina”/REA	Louisiana (USA)	[13]
*Geothlypis trichas*	Common yellowthroat	PCR (blood)	*Rickettsia* sp. “Argentina”/REA	Louisiana (USA)	[13]
*Helmitheros vermivorus*	Worm-eating warbler	PCR (blood)	*Rickettsia* sp. “Argentina”/REA	Louisiana (USA)	[13]
*Pheucticus ludoviacianus*	Rose-breasted grosbeak	PCR (blood)	*Rickettsia* sp. “Argentina”/REA	Louisiana (USA)	[13]
*Oporomis formosus*	Kentucky warbler	PCR (blood)	*Rickettsia* sp. “Argentina”/REA	Louisiana (USA)	[13]
*Catharus ustulatus*	Swainson’s thrush	PCR (blood)	*Rickettsia* sp. “Argentina”/REA	Louisiana (USA)	[13]
*Melospiza georgiana*	Swamp sparrow	PCR (blood)	*Rickettsia* sp. “Argentina”/REA	Louisiana (USA)	[13]
*Empidonax virescens*	Acadian flycatcher	PCR (blood)	*Rickettsia* sp. “Argentina”/REA	Louisiana (USA)	[13]
*Erithacus rubecula*	Robin	PCR (blood)	*Rickettsia helvetica*	Hungary	[14]
*Prunella modularis*	Dunnock	PCR (blood)	*Rickettsia helvetica*	Hungary	[14]
*Columba livia domestica*	Feral pigeon	PCR (spleen)	*Rickettsia* sp.	Italy	[15]
*Aptenodytes patagonicus*	King penguin	Smear (blood)	*Rickettsia*-like	South Africa	[16]
*Phasianus colchicus*	Pheasant	IFI (blood serum)	SFG *Rickettsia* sp.	Italy	[17]
*Struthio camelus*	Ostrich	IFI (blood serum)	SFG *Rickettsia* sp.	Zimbabwe	[18]

Legend. IFI: indirect immunofluorescence test; PCR: polymerase chain reaction; REA: *Rickettsia* endosymbiont of *Amblyomma maculatum;* SFG: spotted fever group.

**Table 2:** First description of avian *Babesia* spp. (modified from Pierce, 2000).

Bird Family	Test	*Babesia* Species	Geographical Distribution	References
Accipitridae	Microscopy	*Babesia moshkovskii*	Tadjikistan, Pakistan	[53,54]
Alaudidae	Microscopy	*Babesia kazachstanica*	Kazachstan	[69,70]
Apterygidae	Microscopy	*Babesia kiwiensis*	New Zealand	[55]
Ardeinae	Microscopy	*Babesia ardeae*	Indochina	[71]
Corvidae	Microscopy	*Babesia frugilegica*	Kazachstan, Pakistan	[69,70]
Emberyzinae	Microscopy	*Babesia emberizica*	Kazachstan	[69,70]
Falconidae	Microscopy	*Babesia shortti*	Egypt, Italy	[72,73]
Gruidae	Microscopy	*Babesia balearicae*	Egypt	[70,72]
Hirundinidae	Microscopy	*Babesia rustica*	Kazachstan, Kenya	[69,70]
Laridae	Microscopy	*Babesia bennetti*	Spain	[74]
Passeridae	Microscopy	*Babesia mujunkumica*	Kazachstan	[69,70]
Spheniscidae	Microscopy	*Babesia peircei*	South Africa	[75]
Sulidae	Microscopy	*Babesia poelea*	Sand Island, Johnston Atoll, Central Pacific	[76]
Upupidae	Microscopy	*Babesia krylovi*	Kazachstan	[69,70]
Alcidae	Microscopy and PCR	*Babesia uriae*	California (US)	[56]
Phalacrocidae	Microscopy	*Babesia ugwidiensis*	South Africa	[57]

## Data Availability

The data presented in this study is contained within the article.
